# Relative Fat Mass as an Estimator of Abdominal Adiposity in Youth Across the BMI Spectrum from Normal Weight to Obesity

**DOI:** 10.1111/ijpo.70059

**Published:** 2025-10-15

**Authors:** Wonhee Cho, Joon Young Kim, Silva Arslanian

**Affiliations:** ^1^ Department of Exercise Science, David B. Falk College of Sport Syracuse University Syracuse New York USA; ^2^ Center for Pediatric Research in Obesity and Metabolism and the Division of Pediatric Endocrinology, Diabetes and Metabolism University of Pittsburgh, School of Medicine, UPMC Children's Hospital of Pittsburgh Pittsburgh Pennsylvania USA

**Keywords:** abdominal adiposity, obesity, relative fat mass, total body adiposity, youth

## Abstract

**Background:**

Relative fat mass (RFM) has been used to estimate whole‐body fat percentage (%BF). However, whether RFM can mirror abdominal adiposity is unknown.

**Objective:**

In youth, we examined if RFM reflects abdominal adiposity, if there are age/race/sex differences in the relationships between RFM and abdominal adiposity, and if our present‐youth‐cohort‐derived RFM (RFM‐Y) improves the prediction of %BF and abdominal adiposity.

**Methods:**

In 358 youth (aged 10–19 years; 168 black/198 white) with normal weight and overweight/obesity (without secondary or syndromic obesity), visceral (VAT), subcutaneous (SAT) and total abdominal adipose tissue (TAT) were assessed by MRI/CT and %BF with DXA. RFM was calculated for ages 10–14 years as [74 − (22 × height/waist circumference [WC]) + (5 × sex)] and 15–19 years as [64 − (20 × height/WC) + (12 × sex)]; sex = 0 for boys, 1 for girls. To develop and validate RFM‐Y, linear regression, concordance correlation coefficient, Bland–Altman plots and intraclass correlation coefficient were conducted.

**Results:**

RFM associated with VAT, SAT and TAT (*r* = 0.66, 0.79, 0.79, respectively; all *p* < 0.001). The slope was significantly greater in (a) younger versus older youth for VAT, SAT and TAT; (b) whites versus blacks for VAT; and (c) girls versus boys for SAT and TAT. RFM‐Y versus traditional RFM showed improved predictive ability for %BF (*R* ^2^ = 0.83 vs. 0.77) and abdominal adiposity (*R* ^2^ ranging from 0.51 to 0.80 vs. 0.37 to 0.63).

**Conclusions:**

RFM reflects abdominal adiposity besides %BF and could be used in longitudinal/interventional youth trials, obviating the need for expensive imaging to assess changes in total and abdominal adiposity.

## 1 Introduction

Against the backdrop of the obesity epidemic in youth [1], total body and abdominal adiposity are major risk factors for youth‐onset cardiometabolic disorders, including insulin resistance, type 2 diabetes, dyslipidemia, hypertension and metabolic dysfunction associated with steatotic liver diseases [2, 3, 4, 5]. A reliable and clinically acceptable measure of total body adiposity is dual‐energy X‐ray (DXA), which provides information on total body fat mass (BF), %BF and fat free mass (FFM) [6]. With respect to abdominal adiposity, magnetic resonance imaging (MRI) and computed tomography (CT) provide accurate measures for abdominal visceral (VAT), subcutaneous (SAT) and total adipose tissue (TAT) [7]. Recent efforts have been made, though, to develop various equations to estimate %BF in adult populations [8, 9, 10, 11], to preclude the need for imaging‐based measures of body composition when DXA facilities are not available in multicenter global observational and/or interventional studies. However, previous equations require various measurements and variables (e.g., race and skinfold measures) and/or relatively complex calculations, indicating limited utility for clinical and epidemiological studies [12, 13].

To reduce the burdens of measurement, cost and complex equations, relative fat mass (RFM) and RFM paediatric (RFMp) were recently introduced as simple and reliable surrogates of whole‐body fat% in adults [12] and youth [13], respectively. From here on, we will refer to RFM and RFMp as ‘RFM’ collectively for simplicity purposes. Previous studies showed that cardiometabolic risk factors are more prevalent in youth with abdominal obesity than in those with general obesity [14, 15, 16, 17, 18]. Considering that the equations for RFM include waist circumference (WC), a strong predictor for total and abdominal adiposity [4] and a major risk factor for dysmetabolic syndrome in youth [16, 17, 18], we hypothesised that RFM could reflect abdominal (visceral and subcutaneous) adiposity in addition to total body adiposity. To our knowledge, studies investigating the association between RFM and imaging‐based abdominal adiposity in youth remain limited or non‐existent. Therefore, we aimed to investigate (1) whether RFM can estimate abdominal adiposity measured by MRI/CT in youth with normal weight and obesity, (2) whether the relationship between RFM and imaging‐based measures of abdominal adiposity differs by age, race and sex in youth and (3) whether a newly developed equation based on our present youth cohort‐derived RFM (RFM‐Y), specific to black and white youth aged 10–19 years, can enhance the prediction of total and abdominal adiposity in youth.

## 2 Methods

### 2.1 Participants

A total of 358 youth (172 boys/186 girls; 168 black/190 white) with normal weight (*n* = 88) and overweight/obesity (36 overweight [body mass index (BMI) ≥ 85th–< 95th percentile for age and sex] and 234 with obesity [BMI ≥ 95th percentile]) were included in this cross‐sectional study. To simplify, we will refer to participants with overweight in the current study as having ‘obesity’. Inclusion criteria of the current study were youth 10–19 years old, at Tanner Stage II–V puberty, and who were self‐reported as white or black. Exclusion criteria were secondary or syndromic obesity, pre‐existing systemic or psychiatric chronic conditions, intake of medications that influence carbohydrate or lipid metabolism, or missing variables of interest, including age, sex, weight, height, WC, hip circumference, total and abdominal adiposity. As such, of 675 participants enrolled, we excluded 96 participants aged < 10 or ≥ 20 years old or Tanner 1 stage. We further excluded 49 participants with secondary or syndromic obesity, pre‐existing systemic or psychiatric chronic conditions or intake of medications that influence carbohydrate or lipid metabolism. Additionally, we excluded 172 participants who did not have variables of our interest in the present study. Statistical power calculations were not conducted prior to the study, as this was a secondary analysis from an existing dataset. However, we conducted a post hoc power analysis, which demonstrated that the current sample size (*N* = 358) provided more than 99% statistical power for the multiple linear regression analysis to examine associations between predictor variables and abdominal adiposity measures. The study was approved by the institutional review board of the University of Pittsburgh and written informed parental consent and child agreement were obtained from all participants.

### 2.2 Study Procedures and Measurements

Study participants were recruited from March 1997 through October 2012 via the general media, including newspaper advertisements, flyers posted on the medical campus and city bus, and through outpatient clinics in the Weight Management and Wellness Center and the Division of Paediatric Endocrinology. All participants underwent medical history and physical examination at the Paediatric Clinical and Translational Research Center of Children's Hospital of Pittsburgh. Pubertal development was assessed by physical examination, performed by a paediatric endocrinologist and/or a certified nurse practitioner, using Tanner criteria [19]. Evaluation for secondary or syndromic obesity was conducted by board‐certified paediatric endocrinologists or a certified nurse practitioner based on the participant's medical history, symptoms and physical examination. Height and weight were measured to the nearest 0.1 cm and 0.1 kg, respectively, with a fixed wall stadiometer (Holtain Ltd., Crosswell, UK) and a standard balanced scale, to compute BMI (kg/m^2^) and BMI *z*‐score. WC (cm) was taken at the midpoint between the lowest rib and the iliac crest, using a spring‐loaded Gulick tape. Hip circumference (cm) was assessed at the widest part of the buttocks using the same tape. These measurements were obtained by a certified nurse practitioner, and the average value of two measurements was used. Total BF, FFM and %BF were measured by lunar iDXA (GE Healthcare, Madison, WI), and abdominal adiposity including VAT, SAT and TAT (all cm^2^) were assessed by either CT (*n* = 212) at the L_4–5_ intervertebral space or MRI (*n* = 146) as formerly described [7, 20]. TAT was computed as the sum of SAT and VAT. The transition from CT to MRI was mandated by the study section as part of the competitive grant renewal process. Klopfenstein et al. [21] reported a strong correlation (*r* = 0.89–0.95) and good agreement between CT and MRI for quantifying abdominal adiposity in a cohort of individuals aged 18–43 years with BMI of 24–49 kg/m^2^ who underwent both MRI and CT scans on the same day. Additionally, we confirmed no significant differences in key demographic and adiposity parameters (age, weight, BMI and %BF) between participants who underwent MRI versus CT, and regression analyses yielded consistent results with the total cohort, indicating no bias from imaging methodology (Table S1).

### 2.3 Calculation for RFM Index

RFM for youth aged 10–14 years was calculated as published: [74 − (22 × height/WC) + (5 × sex)] [13] and for youth aged 15–19 years calculated using the same equation as before for adults: [64 − (20 × height/WC) + (12 × sex)] [12]. In both equations, sex = 0 for boys and 1 for girls was inputted. Woolcott et al. [12] validated the adult RFM formula for youth aged 15–19 years as showing reasonable estimates of %BF, with coefficients of determination (*R* ^2^) of 0.79 for boys and 0.72 for girls, respectively.

### 2.4 Statistical Analysis

Three‐way ANOVA for continuous variables and a chi‐square test for categorical variables were conducted to compare descriptive characteristics by age, race (whites vs. blacks) and sex (boys vs. girls) in the total cohort. For Tanner stage, participants were categorised as early pubertal (Tanner stage II/III) and late‐pubertal (Tanner stage IV/V) for the present analysis. To address objectives 1 and 2, general linear models were conducted to examine age‐, race‐ and sex‐specific effects using multivariate regression analyses between independent (RFM) and dependent (VAT, SAT and TAT) variables. Main effects (age, race, sex and RFM), two‐, three‐ and four‐factor interaction effects (e.g., age × race × sex × RFM) for each abdominal adiposity were tested in multiple regression analyses. If the four‐factor interaction effect or three‐factor interactions of [age × race × RFM] or [race × sex × RFM] were significant, additional multiple regression analyses were performed separately within each race group to examine age‐ and sex‐specific differences in slopes and intercepts (Figure S1). If the four‐factor interaction effect was not significant, the model was reduced to examine the three‐factor interactions of [age × race × RFM], [age × sex × RFM] or [race × sex × RFM]. If the three‐factor interaction effect was not significant, main effects and two‐factor interaction analyses were conducted to examine age‐, race‐ and sex‐specific differences in slopes and intercepts. Furthermore, additional multiple regression analyses stratified by weight status (normal weight vs. obesity) and Tanner stage (II–III vs. IV–V) were conducted to examine potential subgroup differences (Figures S2 and S3). The Bland–Altman analysis was used to assess concordance between RFM and DXA‐measured %BF in the total cohort and across age, race and sex groups [22]. This analysis included the Limits of Agreement (LOA), which describes the mean difference (bias) between two methods and their 95% Confidence Interval (CI).

To address objective 3, we developed the prediction equation for our RFM‐Y from the current youth cohort data. We initially categorised all the participants randomly into two independent datasets, with 75% (*n* = 268) assigned to the development dataset and 25% (*n* = 90) to the validation dataset, using a random number generator. The development dataset was used to develop the equation, and the validation dataset was utilised for validating our new RFM‐Y equation. Using the development dataset, demographic and anthropometric variables with *r* > 0.2 with DXA‐measured %BF were included in forward multiple stepwise linear regression analysis to predict %BF as a new RFM equation (Table S2). For validation, we calculated the predicted %BF using the developed anthropometric equations for the validation dataset participants. Subsequently, we compared our RFM‐Y with DXA‐measured %BF in the validation dataset for cross‐validation. The dependent samples *t*‐test was further used to check the difference between the RFM‐Y and %BF. Additionally, we conducted regression analyses to compare *R* ^2^, mean squared error (MSE) and standard of error estimate (SEE) of the fitted models from traditional RFM and RFM‐Y equation in the validation dataset. Moreover, Lin's concordance correlation coefficient (CCC), intraclass correlation coefficient (ICC) and Bland–Altman plots were used to evaluate the agreement between RFM‐Y and DXA‐measured %BF in the validation dataset. Multicollinearity was evaluated by tolerance value and variance inflation factor (VIF). A tolerance value > 0.1 or a VIF < 10 for each independent variable was considered the absence of significant multicollinearity. The Breusch–Pagan test was performed to examine potential heteroscedasticity. Lastly, we used regression analyses to compare the relationships of imaging‐based abdominal adiposity (VAT, SAT and TAT) with traditional RFM and our RFM‐Y by examining differences in the *R* ^2^ and SEE of the fitted models in the total cohort. All statistical analyses were analysed using SPSS 29 version (SPSS Inc., Illinois, USA) and GraphPad Prism 10.4.1 (GraphPad Software Inc., California, USA). Data are mean ± standard deviation (SD) with *p* ≤ 0.05.

## 3 Results

### 3.1 Participants' Characteristics by Age, Race and Sex

Table 1 depicts participants' characteristics. The main effect of age showed no significant differences in the BMI *z*‐score, %BF, VAT or RFM (all *p* > 0.05). There were no significant differences in descriptive characteristics between black and white youth, except for lower VAT in blacks (*p* = 0.002). Age, BMI, BMI *z*‐score, WC, Total BF and VAT were similar between boys and girls (all *p* > 0.05). However, weight, height and FFM were significant by sex, higher in boys versus girls, and Tanner stage and DXA‐measured %BF, and hip circumference, CT/MRI‐measured SAT and TAT were significantly higher in girls versus boys (all *p* < 0.05). Consistent with DXA‐measured %BF, calculated RFM was significant by sex, higher in girls versus boys (*p* < 0.001).

**TABLE 1:** Descriptive characteristics of participants.

	Whites (*n* = 190)		Blacks (*n* = 168)		Main effect		
	Boys (*n* = 92)	Girls (*n* = 98)	Boys (*n* = 80)	Girls (*n* = 88)	*p* by age	*p* by race	*p* by sex
Age (years)	14.3 ± 1.7	14.7 ± 2.1	14.4 ± 1.8	14.1 ± 2.0	—	0.352	0.906
Tanner Stage (% II–III/IV–V)	42/58	14/86	31/69	12/88	**< 0.001**	0.401	**< 0.001**
Weight (kg)	81.9 ± 28.7	83.2 ± 26.3	88.8 ± 27.9	79.9 ± 24.0	**< 0.001**	0.952	**0.026**
Height (cm)	167.9 ± 10.4	162.1 ± 7.8	168.5 ± 9.7	160.6 ± 7.1	**< 0.001**	0.722	**< 0.001**
BMI (kg/m^2^)	28.5 ± 8.1	31.4 ± 8.8	31.4 ± 8.1	30.7 ± 8.2	**0.008**	0.639	0.646
BMI *z*‐score	1.5 ± 1.2	1.7 ± 1.0	1.8 ± 1.0	1.7 ± 0.9	0.622	0.696	0.574
WC (cm)	94.8 ± 20.6	94.5 ± 19.0	96.5 ± 19.7	92.8 ± 19.8	**0.015**	0.672	0.071
HC (cm)	98.7 ± 16.4	109.1 ± 18.9	102.6 ± 16.4	104.4 ± 17.5	**0.005**	0.949	**< 0.001**
Total BF (kg)	28.3 ± 17.1	35.9 ± 17.1	31.7 ± 18.5	32.5 ± 15.4	**0.018**	0.839	0.733
FFM (kg)	50.4 ± 13.0	43.4 ± 9.0	54.7 ± 11.5	44.4 ± 8.9	**< 0.001**	0.178	**< 0.001**
%BF (%)	32.1 ± 12.3	41.5 ± 9.7	32.7 ± 13.7	38.9 ± 9.3	0.721	0.517	**0.002**
VAT (cm^2^)	62.8 ± 39.8	65.2 ± 32.4	54.3 ± 37.3	44.4 ± 23.4	**0.04**	**0.002**	0.087
SAT (cm^2^)	322.2 ± 226.4	438.8 ± 234.1	373.5 ± 251.7	381.9 ± 217.8	**0.008**	0.741	**0.02**
TAT (cm^2^)	385.3 ± 261.3	504.0 ± 261.1	427.8 ± 281.1	426.4 ± 235.6	**0.016**	0.463	**0.03**
RFM	31.1 ± 8.2	40.0 ± 7.6	31.6 ± 8.2	39.4 ± 7.8	0.387	0.726	**< 0.001**

*Note*: Data are mean ± standard deviation. Bold *p* values are statistically significant (*p* < 0.05).

Abbreviations: %BF, percent body fat; BMI, body mass index; FFM, fat‐free mass; HC, hip circumference; *p* by age, *p* value for comparison by age in the total cohort; *p* by race, *p* value for comparison by race in the total cohort; *p* by sex, *p* value for comparison by sex in the total cohort.; RFM, relative fat mass; SAT, subcutaneous adipose tissue; TAT, total adipose tissue; total BF, total body fat mass; VAT, visceral adipose tissue; WC, waist circumference.

### 3.2 Relationships Between RFM and Abdominal Adiposity Measures in the Total Cohort

**FIGURE 1:**
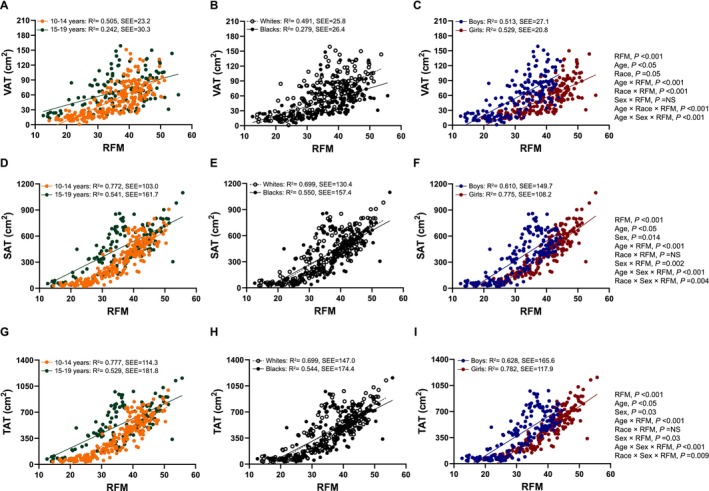
Relationship between calculated relative fat mass (RFM) and CT/MRI‐measured VAT (A–C), SAT (D–F) and TAT (G–I) in the total cohort by age group (Left panel), by race (Middle panel) and by sex (Right panel).

Figure 1 illustrates the significant linear relationships of RFM with VAT, SAT and TAT after consideration of age, race and sex effects in the total cohort (*R* ^2^ = 0.38, 0.63 and 0.63, respectively; all *p* < 0.001). Four‐factor interaction [age × race × sex × RFM] effect was not significant for any of the abdominal adiposity measures (*p* > 0.05). For the relationship between RFM and VAT, there were significant (*p* < 0.05) main effects of age, race and two‐factor [age × RFM and race × RFM] and three‐factor [age × race × RFM and age × sex × RFM] interaction effects (Figure 1A–C). The slope of the regression line between RFM and VAT for younger youth was significantly higher than that for older youth, while the intercept was higher in older youth than for younger youth (Figure 1A). Thus, for a given RFM, older youth had more VAT than younger youth; however, this difference was significantly reduced with increasing RFM (*p* < 0.001). Additionally, the slope and intercept were significantly higher for white youth than for black youth (Figure 1B). White youth had higher VAT than did the black youth for a given RFM, wherein the magnitude of the difference is increased with increasing RFM (*p* < 0.001). For the relationships of RFM with SAT and TAT, there were significant (*p* < 0.05) main effects of age and sex and two‐factor [age × RFM and sex × RFM] and three‐factor [age × sex × RFM and sex × race × RFM] interaction effects (Figure 1D–I), respectively. The slopes of the regression lines of RFM with SAT and TAT were significantly higher in younger versus older youth, while intercepts were higher in older versus younger youth (Figure 1D,G). The slope and intercept were similar in white versus black youth (Figure 1E,H). In addition, the slopes of the regression lines of RFM with SAT and TAT were significantly higher in girls versus boys, while intercepts were higher in boys versus girls (Figure 1F,I). Thus, for a given RFM, older versus younger youth and boys versus girls had a greater increase in SAT and TAT. As RFM increased, the magnitude of these differences decreased in younger versus older youth and in girls versus boys (both *p* < 0.05).

### 3.3 Relationships Between RFM and Abdominal Adiposity Measures by Sex and Age Within Each Race

Within each race, RFM was significantly correlated with all abdominal adiposity measures independent of age and sex (Figure S1). For VAT, in both races, there were similar patterns as those observed in the total cohort, showing a significant main effect of age and two‐factor [age × RFM] and three‐factor [age × sex × RFM] interaction effects on the relationship between RFM and VAT (Figure S1A,B,G,H). However, in black youth, the two‐factor [sex × RFM] interaction effect did not have a significant effect on the relationships of RFM with SAT and TAT. Thus, sex‐related differences in the slopes of regression lines between RFM with SAT and TAT were observed only in white youth (Figure S1I–L). When the participants were stratified by weight status (normal‐weight and obesity) in each group, interaction terms and significance levels were similar in the groups (Figure S2). When they were stratified by Tanner stage (II–III and IV–V), compared with youth of a lower Tanner stage (II–III), those of a higher Tanner stage (IV–V) exhibited more complex interactions, reflecting significant influences of sex and age (both two‐ and three‐factor interactions) on the relationship between RFM and abdominal adiposity (Figure S3). Details of subgroup analyses (by age, sex and race, weight status and Tanner stage) are illustrated in Supporting Information.

### 3.4 Agreement Between RFM and DXA‐Measured Total Body Fat Percentage

**FIGURE 2:**
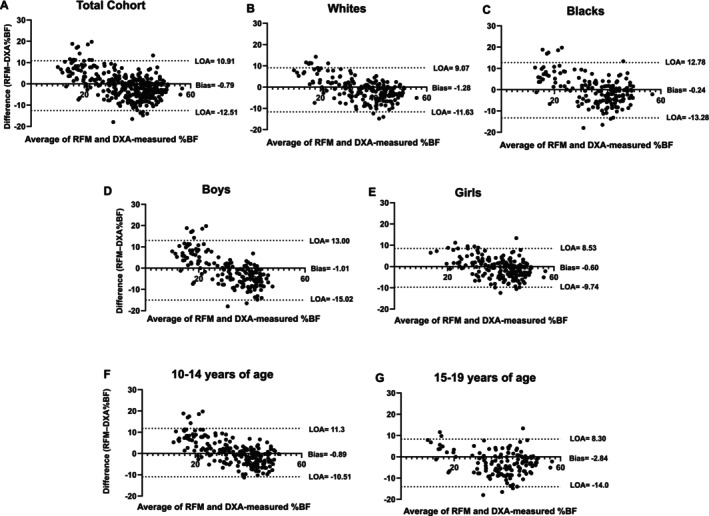
Bland–Altman plots showing the agreement between RFM and DXA‐measured %BF in the total cohort (A), by race (B and C), by sex (D and E) and by age groups (F and G). Two dotted lines represent the 95% limits of agreement (LOA).

In the total cohort, RFM was associated with DXA‐measured total BF and %BF (*R* ^2^ = 0.61, SEE = 10.7 and 0.77, SEE = 5.7; all *p* < 0.001). Moreover, the Bland–Altman analyses confirmed good agreement between %BF and RFM in the total cohort (Figure 2A) and across race (Figure 2B,C), sex (Figure 2D,E) and age (Figure 2F,G). In the total cohort and subgroup analyses, Bland–Altman plots revealed small mean biases between %BF and RFM (all < 1.5%) (Figure 2).

### 3.5 Development and Validation of RFM‐Y Equation

**FIGURE 3:**
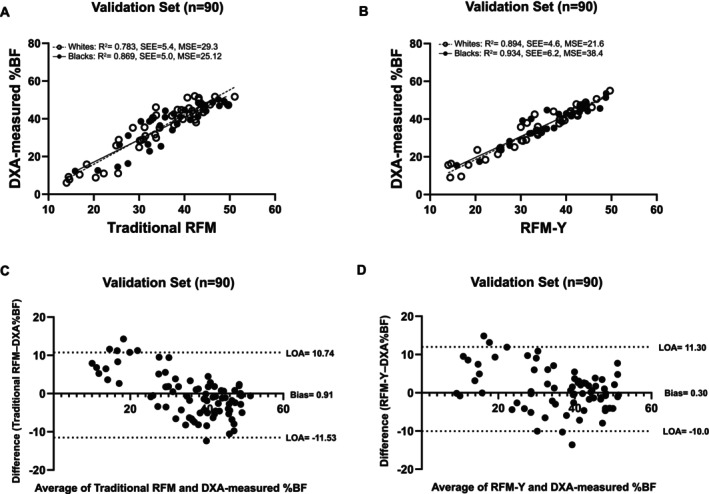
Relationship between DXA‐measured total body fat percentage and predicted by equations (traditional RFM and RFM‐Y) (A and B). Bland–Altman plots showing the agreement between DXA‐measured total body fat percentage and traditional RFM (C) and RFM‐Y (D). Two dotted lines represent the 95% limits of agreement (LOA) (C and D).

**FIGURE 4:**
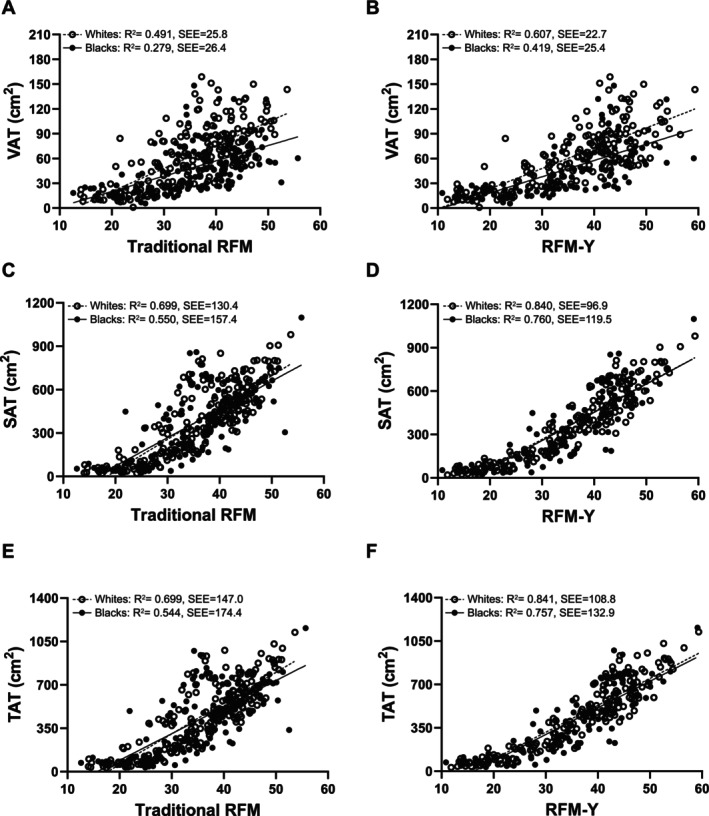
Relationship between imaging‐based abdominal adiposity (VAT, SAT and TAT) and predicted by equations (traditional RFM and RFM‐Y).

We generated our own RFM equation from the present youth cohort data using forward multiple stepwise linear regression analysis. Repeated construction of a regression model allowed automatic selection of independent variables from our dataset. The Breusch–Pagan test for heteroskedasticity showed that the assumption of constant variance of error terms is met for the total cohort and across race, sex and age groups (all *p* > 0.8). Additionally, all tolerance values exceeded 0.58 and all VIF values were less than 1.78, indicating no significant multicollinearity. Our final equation is as follows: RFM‐Y = 96 + (−31.7 × height/WC) + (5.5 × sex) + (−23.6 × WC/hip circumference) + (−0.65 × age) + (0.12 × height). When the RFM‐Y equation was further evaluated in the validation dataset, there was no significant difference against DXA‐measured %BF in the total cohort and across races (all *p* > 0.05), indicating good agreement. Figure 3 shows the relationships of traditional RFM (Figure 3A) and our RFM‐Y (Figure 3B) with %BF in the validation dataset. Compared with traditional RFM equations, the RFM‐Y equation demonstrates a more linear relationship with %BF in white and black youth (Figure 3B). Lin's CCC and the ICC analyses indicated robust agreement between RFM‐Y and DXA‐measured %BF in the validation dataset in both racial groups. Among black youth, Lin's CCC was 0.898 (95% CI: 0.80–0.95) and the ICC was 0.939 (95% CI: 0.91–0.96). Among white youth, Lin's CCC was 0.885 (95% CI: 0.81–0.93) and ICC was 0.966 (95% CI: 0.95–0.98). Further, Bland–Altman plots for traditional RFM (Figure 3C) and RFM‐Y (Figure 3D) against %BF demonstrate good agreement, with RFM‐Y exhibiting a smaller mean bias (mean bias = 0.3) compared to traditional RFM (mean bias = 0.9), indicating improved accuracy. Figure 4 shows the relationship of imaging‐based abdominal adiposity with traditional RFM (Figure 4A,C,E) and our RFM‐Y (Figure 4B,D,F) in the total cohort. Compared with the traditional RFM equation, RFM‐Y shows a more robust relationship with VAT (Figure 4B), SAT (Figure 4D) and TAT (Figure 4F), indicating the tighter fit between abdominal adiposity and RFM‐Y versus traditional RFM.

## 4 Discussion

The current study demonstrates that: (1) RFM derived from the equations developed by Woolcott et al. [12, 13] reflects not only total body adiposity but also abdominal adiposity in youth with normal weight and obesity, and (2) there are age‐, race‐ and sex‐specific differences in the relationships between RFM and abdominal adiposity. Furthermore, using our present youth cohort, we developed and validated a single equation for RFM (RFM‐Y) that robustly predicts whole‐body fat percentage as well as abdominal adiposity.

To date, despite the valuable demonstration of the use of RFM in assessing %BF, there are no paediatric data regarding the relationship of RFM with abdominal adiposity. Studies in adults showed strong relationships of RFM with %BF (*R* ^2^ = 0.73 to 0.84) [12, 23] and trunk‐fat% (*R* ^2^ = 0.79) [24] by DXA. In youth, a study of 10 390 boys and girls aged 8–19 years exhibited *R* ^2^ of 0.74–0.79 for the relationship of RFM with %BF [13]. Consistently, our data revealed a strong relationship of RFM with total BF and %BF (*R* ^2^ ranging from 0.61–0.86) in youth across the spectrum of BMIs, supporting from the single previous paediatric literature [13]. Moreover, our Bland–Altman analyses confirmed good agreements between RFM and DXA‐measured %BF across age, race and sex groups, supporting the potential use of RFM as a practical surrogate for adiposity measurement. Additionally, and for the first time, our study displays robust associations of RFM with abdominal adiposity including VAT, SAT and TAT independent of age, race and sex (Figure 1). Abdominal adiposity is highly associated with cardiometabolic risk [16, 17, 18, 25, 26]. In a study of 28 youth with obesity (mean age: 13 years), half of them were insulin‐resistant while the other half were insulin‐sensitive, the insulin‐resistant group exhibited higher abdominal adiposity, despite being matched for body composition suggesting a specific link between increased abdominal fat and the early development of insulin resistance [25]. Moreover, irrespective of race, increased visceral adiposity imparts significant health risks. Black and white youth with high versus low VAT, measured by CT scan, had lower in vivo insulin sensitivity despite similar BMI and total body adiposity, in addition to having elevated blood pressure [27]. Furthermore, black youth with high VAT versus low VAT demonstrated increased diabetogenic risk while white youth with high versus low VAT exhibited increased atherogenic risk [27]. Against this backdrop of abdominal adiposity foretelling the heightened health risk in adolescents with obesity, a simple measure of abdominal adiposity, such as calculated RFM, that can be used repeatedly in longitudinal therapeutic interventions may enhance research of obesity in youth. Thus, based on our findings, RFM could be utilised as an estimator of abdominal adiposity besides total body adiposity in youth, bypassing the need for expensive and labor‐intensive imaging methodologies.

Our comparisons of regression analyses by age, race and sex provide novel information to the paediatric literature. We found significant age‐, race‐ and sex‐specific differences in the relationship between RFM and abdominal adiposity for a given measure of RFM. Our finding that older youth have greater abdominal adiposity is consistent with previous research demonstrating that abdominal adiposity increases progressively with age from childhood (5 to 17 years) into adulthood [28]. However, our analyses further showed that the slopes of the regression line between RFM and abdominal adiposity were significantly steeper in younger versus older youth (Figure 1A,D,G), implying that the gap between the two slopes, younger versus older, became narrow potentially attributable to the reduced rate of increment in abdominal fat as shown previously in longitudinal observations over 3 to 5 years in black and white youth (mean age: 8.1 ± 1.6 years at baseline) [29].

We further observed race‐specific differences in the relationship between RFM and VAT, with a greater slope in white versus black youth. In this case, a larger gap between the two regression lines (whites vs. blacks) was observed at the increased level of RFM, primarily due to our and others' previous observations of higher VAT in white versus black youth [20, 27, 30, 31]. Moreover, our data showed race‐related differences in the strength of the relationships, with stronger associations in whites versus blacks between RFM and TAT and SAT, but not VAT. Taken together, our observation of RFM estimating VAT reliably in white and black youth with similar strength of relationship suggests that RFM can be utilised as an estimator of abdominal adiposity.

Lastly, we found that girls versus boys have a stronger relationship between RFM and SAT and TAT. This might be because girls tend to have a higher proportion of SAT or TAT than boys [7, 32, 33], amplifying the relationship signals. Our observation of a 26% higher RFM in girls versus boys mirrors imaging‐based results showing 19% higher SAT and 15% higher TAT, despite similar WC (a main component of RFM equations) between the two groups. Collectively, these findings suggest that RFM might better reflect abdominal fat in girls than in boys, despite both showing significant and strong correlations.

Another novelty of the current study is that we developed and validated a single equation, the RFM‐Y, with improved prediction for estimating %BF across age, race and sex. RFM‐Y expands on the traditional RFM by incorporating additional anthropometric measures, including hip circumference, age and height as independent predictors. Inclusion of hip circumference improves the accuracy and clinical relevance of the model. Previous literature has demonstrated that hip circumference is strongly correlated with total fat surface area, as measured by MRI, in both early and late pubertal girls (*r* = 0.79–0.97) [34]. Another study of 452 Chinese youth aged 6–9, hip circumference showed a significant predictor of DXA‐measured abdominal obesity (abdominal fat percentage ≥ 85th percentile), with an area under the curve ranging from 0.87 to 0.88 [35]. Moreover, RFM‐Y provides a single formula applicable across different youth populations with a wide range of ages (10–19 years), making it more practical for broader use without the need for age‐specific equations. Compared with the traditional RFM equations, our new RFM‐Y equation showed a stronger linear relationship with DXA‐measured %BF in both black and white youth (Figure 3B), suggesting a stronger fit with total adiposity compared with traditional RFM. Moreover, the RFM‐Y equation demonstrated better agreement with %BF than traditional RFM, as indicated by its lower mean bias, improved accuracy, and reduced systematic error in estimating %BF (Figure 3D). Additionally, the new RFM‐Y equation showed a tighter linear relationship with VAT (Figure 4B), SAT (Figure 4D) and TAT (Figure 4F) in both black and white youth compared with traditional RFM. These findings suggest that the RFM‐Y equation provides a value‐added method for total body and abdominal adiposity estimation in youth populations.

The strengths of this investigation include (1) a first‐time assessment of the relationship between RFM and imaging‐based abdominal adiposity in youth with normal weight and obesity, (2) comparisons of the regression analysis between RFM and abdominal adiposity by age, race and sex, (3) the development of a new RFM‐Y equation applicable to the youth ages 10 to 19 years old, (4) a large youth sample to develop and validate anthropometric equations for a new RFM‐Y and (5) a well‐balanced representation of boys and girls (48% vs. 52%) and blacks and whites (47% vs. 53%). A potential limitation would be that our analysis was limited to black and white youth, thereby hindering external validity and generalisation of our findings to other racial/ethnic groups. An additional limitation is that our new RFM‐Y equation was developed and validated within the same cohort, which may affect the generalisability of our findings. Therefore, a further external validation study is needed to confirm the utility of RFM‐Y across broader populations. Another weakness could be that our study was cross‐sectional in design; thus, causal relationships between RFM and abdominal adiposity cannot be established. Additionally, the present analyses did not include menarche data as a covariate to evaluate its potential influence on body fat composition, relying instead on Tanner stage data for overall pubertal development. Given that Tanner staging provides a general measure of pubertal status, we believe that the absence of specific menarche information is unlikely to have a significant impact on our findings. Furthermore, another limitation is the lack of adjustment for potential clinical confounders, including physical activity, dietary intake and socioeconomic status, as these variables were not available in the dataset. To date, two prospective studies in adults showed that RFM is strongly associated with new‐onset type 2 diabetes [36] and all‐cause and cardiovascular mortality [24]. Longitudinal studies in youth are warranted to determine if there is an important relationship between RFM and obesity‐related comorbidities over time and whether or not such comorbidities could be alleviated with pharmacotherapeutic interventions that result in weight loss and improved total body and abdominal obesity assessed by RFM.

In summary, our data demonstrate that RFM and RFM‐Y, a simple equation‐based estimate of total body fatness, also reflects abdominal adiposity in youth with normal‐weight and obesity. Our findings support the applicability of the repeated use of RFM and RFM‐Y in large‐scale longitudinal, observational and/or interventional therapeutic trials of youth with obesity.

## Author Contributions

W.C., J.Y.K. and S.A. designed the study and analysed data. W.C. wrote the manuscript. J.Y.K. and S.A. critically reviewed the manuscript, are the guarantors of this work and, as such, had full access to all the data in the study and take responsibility for the integrity of the data and the accuracy of the data analysis.

## Conflicts of Interest

The authors declare no conflicts of interest.

## Supporting information


**Data S1:** ijpo70059‐sup‐0001‐Supinfo.pdf.

## Data Availability

The data that support the findings of this study are available on request from the corresponding author. The data are not publicly available due to privacy or ethical restrictions.
