# Supplementary material for: Relative Fat Mass as an Estimator of Abdominal Adiposity in Youth Across the BMI Spectrum from Normal Weight to Obesity
Source: Pediatr Obes. 2025 Oct 15;20(12):e70059. doi: 10.1111/ijpo.70059 (PMC12590103; doi:10.1111/ijpo.70059)
Supplement: Supplementary file 1 — Data S1: ijpo70059‐sup‐0001‐Supinfo.pdf. [file IJPO-20-e70059-s001.pdf]

Relative Fat Mass as an Estimator of Abdominal Adiposity in Youth Across the BMI Spectrum from Normal Weight to Obesity

Wonhee Cho, MS, MPH,[1] Joon Young Kim, PhD,[1]* Silva Arslanian, MD[2]

[1] Department of Exercise Science, David B. Falk College of Sport, Syracuse University, Syracuse, New York, NY, USA;

[2]Center for Pediatric Research in Obesity and Metabolism and the Division of Pediatric Endocrinology, Diabetes and Metabolism, University of Pittsburgh, School of Medicine, UPMC Children's Hospital of Pittsburgh, Pittsburgh, PA, USA

***Corresponding author:**

Joon Young Kim, PhD

Department of Exercise Science, Syracuse University

430H White Hall, Syracuse, New York, 13244

Tel: 1-315-443-1411, Email: jkim291@syr.edu

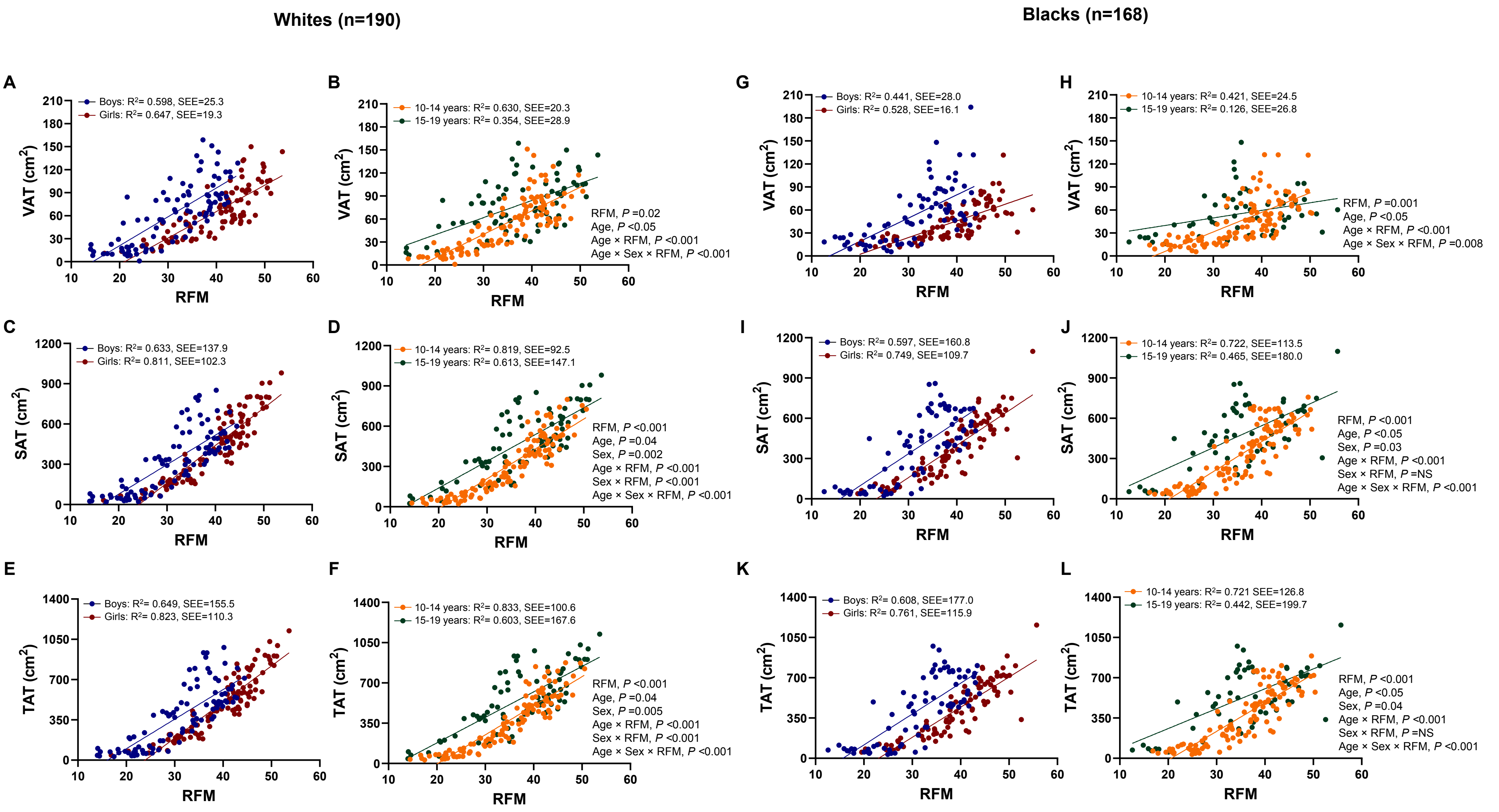

Supplemental Figure 1. Relationships between RFM and abdominal adipose tissue by sex within each race

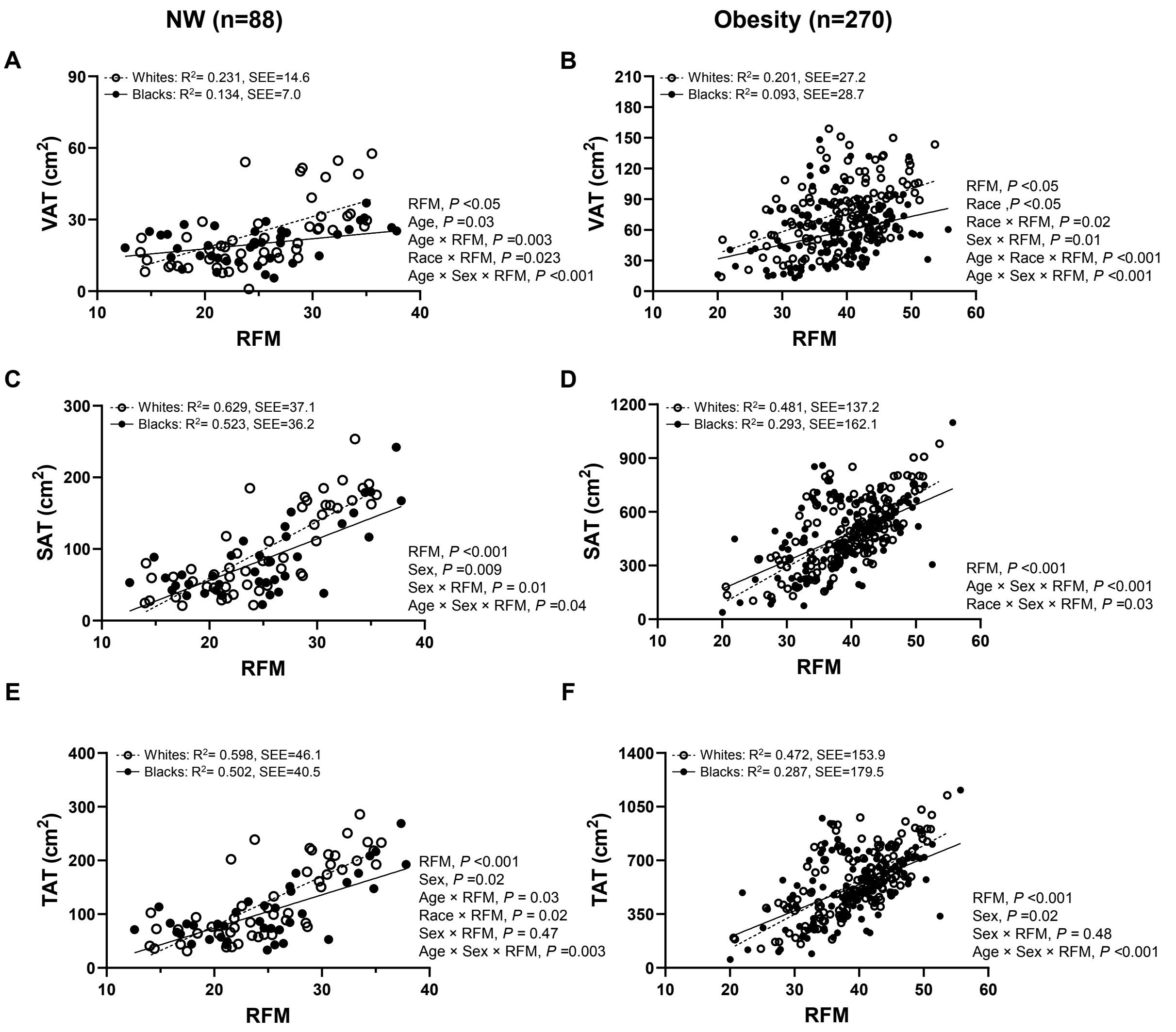

Supplemental Figure 2. Relationships between RFM and abdominal adipose tissue in youth by weight status (normal-weight, n=88; obesity, n=270)

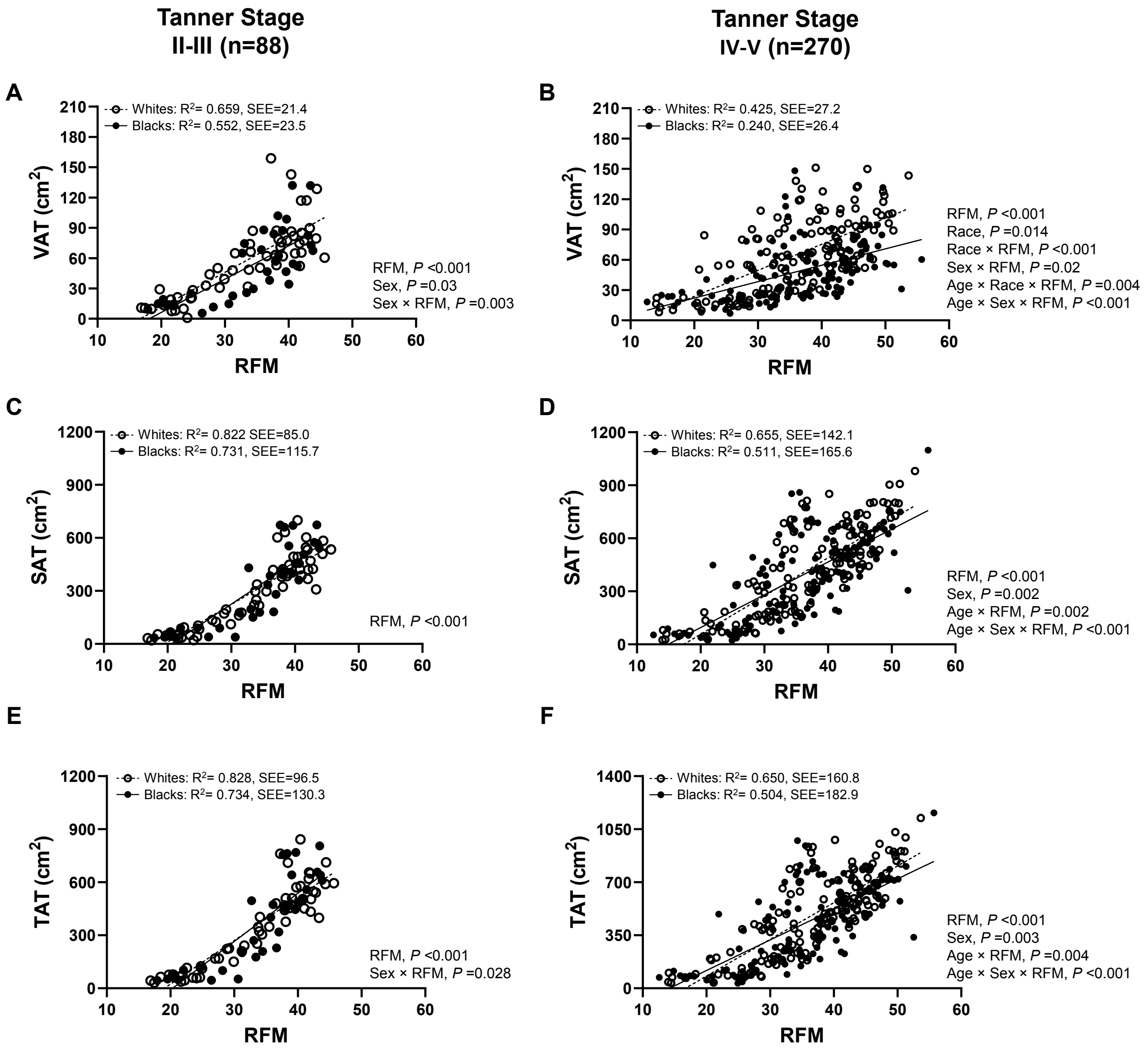

Supplemental Figure 3. Relationships between RFM and abdominal adipose tissue in youth by Tanner Stage (II-III, n=88; IV-V, n=270)

Supplemental Table 1. Descriptive characteristics of participants by imaging methodology

	CT (n=212)	MRI (n=146)	P-value
Age (years)	14.4 ± 2.0	14.4 ± 1.8	0.87
Sex (% boy/girl)	43/57	54/46	0.03
Tanner Stage (% II-III/IV-V)	28/72	20/80	0.1
Weight (kg)	83.6 ± 28.1	82.9 ± 24.9	0.83
Height (cm)	163.5 ± 9.6	166.3 ± 8.9	0.01
BMI (kg/m^2)	30.9 ± 8.8	29.7 ± 7.6	0.15
BMI z-score	1.67 ± 1.1	1.65 ± 1.0	0.79
WC (cm)	94.9 ± 19.6	94.1 ± 19.9	0.71
Total BF (kg)	32.9 ± 18.3	32.1 ± 15.4	0.32
FFM (kg)	47.1 ± 11.4	48.1 ± 11.8	0.1
%BF (%)	36.9 ± 12.9	36.0 ± 10.4	0.48
**RFM**	36.6 ± 9.0	34.4 ± 8.6	0.08

Data are mean ± standard deviation. BMI, body mass index; WC, waist circumference; total BF, total body fat mass; FFM, fat-free mass; %BF, percent body fat; RFM, relative fat mass

Supplemental Table 2. Correlations between percent body fat and demographic and anthropometric variables in development set (n=268)

Variables	r
Age	0.279**
Sex	0.379*
Height	0.189*
Weight	0.739**
Waist Circumference	0.788**
Hip Circumference	0.847**
Waist to Hip ratio	0.293**
Height to Waist ratio	-0.834**

*$P < 0.05$, **$P < 0.01$
